# Using behavior and eye-fixations to detect feigned memory impairment

**DOI:** 10.3389/fpsyg.2024.1395434

**Published:** 2024-09-20

**Authors:** Filomena Gomes, Inês Ferreira, Bruno Rosa, Ana Martins da Silva, Sara Cavaco

**Affiliations:** ^1^Neuropsychology Service, Centro Hospitalar Universitário de Santo António, Porto, Portugal; ^2^Laboratory of Neurobiology of Human Behavior, Centro Hospitalar Universitário de Santo António, Porto, Portugal; ^3^UMIB - Unit for Multidisciplinary Research in Biomedicine, ICBAS - School of Medicine and Biomedical Sciences, University of Porto, Porto, Portugal; ^4^ITR - Laboratory for Integrative and Translational Research in Population Health, Porto, Portugal; ^5^Department of Neurology, Centro Hospitalar Universitário de Santo António, Porto, Portugal

**Keywords:** malingering, novelty preference, familiarity preference, eye-tracking, performance validity tests

## Abstract

**Background:**

Detecting invalid cognitive performance is an important clinical challenge in neuropsychological assessment. The aim of this study was to explore behavior and eye-fixations responses during the performance of a computerized version of the Test of Memory Malingering (TOMM-C) under standard vs. feigning conditions.

**Participants and methods:**

TOMM-C with eye-tracking recording was performed by 60 healthy individuals (31 with standard instruction – SI; and 29 were instructed to feign memory impairment: 21 Naïve Simulators – NS and 8 Coached Simulators – CS) and 14 patients with Multiple Sclerosis (MS) and memory complaints performed. Number of correct responses, response time, number of fixations, and fixation time in old vs. new stimuli were recorded. Nonparametric tests were applied for group comparison.

**Results:**

NS produced fewer correct responses and had longer response times in comparison to SI on all three trials. SI showed more fixations and longer fixation time on previously presented stimuli (i.e., familiarity preference) specially on Trial 1, whereas NS had more fixations and longer fixation time on new stimuli (i.e., novelty preference) specially in the Retention trial. MS patients produced longer response time and had a different fixation pattern than SI subjects. No behavioral or oculomotor difference was observed between NS and CS.

**Conclusion:**

Healthy simulators have a distinct behavioral and eye-fixation response pattern, reflecting a novelty preference. Oculomotor measures may be useful to detect exaggeration or fabrication of cognitive dysfunction. Though, its application in clinical populations may be limited.

## Introduction

Malingering is the volitional feigning or exaggeration of neurocognitive symptoms for the purpose of obtaining material gain or services or avoiding formal duty, responsibility, or undesirable outcome (Slick et al., 1999; Sherman et al., 2020). The detection of noncredible cognitive performance is an important clinical challenge in neuropsychological assessment. The presence of an external incentive (e.g., Social Security benefits, insurance compensation) is an important element to consider when distinguishing people with credible cognitive impairment from feigned cognitive deficits, even in clinical, non-forensic settings. The presence of an external incentive does not necessarily indicate unreliable neuropsychological test performance. However, it has been demonstrated that being in the process of applying for Social Security disability benefits increases the likelihood of noncredible performance (Schroeder et al., 2022; Horner et al., 2023). It has been estimated that between one third to two thirds of clinically referred patients with Social Security disability as an external incentive produce invalid data on performance validity tests - PVTs (Chafetz and Biondolillo, 2013; Schroeder et al., 2022), whereas less than one tenth of low-functioning Child Protection claimants who are motivated to do well failed on PVTs. Frequently, patients referred for routine clinical neuropsychological evaluation utilize the results of the examination for Social Security documentation.

PVTs are objective tests designed to detect invalid cognitive performance, i.e., feigned and/or exaggerated diminished capability (Sweet et al., 2021). PVTs usually require little effort or ability, as they typically are normally performed by a wide range of patients who have *bona fide* neurologic, psychiatric, or developmental problems (Heilbronner et al., 2009).

Most PVTs are forced-choice memory recognition tests and only explore accuracy to detect poor cognitive effort or malingering. Recent studies suggest that response time (Bolan et al., 2002; Kanser et al., 2019; Patrick et al., 2021b) and eye-tracking measures (Heaver and Hutton, 2011; Kanser et al., 2020; Tomer et al., 2020; Patrick et al., 2021a) may produce incremental information to the conventional accuracy responses on PVTs.

The Test of Memory Malingering (TOMM; Tombaugh, 1996) is one of the most widely used PVTs in research and clinical practice. TOMM is a forced-choice visual memory recognition test and the number of correct responses is the standard measure to discriminate between true memory impairment from noncredible performance. Response times are also able to detect feigned memory impairment on TOMM (Bolan et al., 2002; Kanser et al., 2019). The oculomotor behavior during the performance of TOMM has yet to be investigated.

This study aimed to quantify the potential information gains provided by eye fixation data in addition to behavioral response (i.e., response accuracy and response time), in the performance of a computerized version of TOMM (TOMM-C), to distinguish simulators from non-simulators. The clinical applicability of these measures was also explored in a sample of patients with multiple sclerosis and memory complaints. We hypothesized that eye-tracking metrics, in particular eye-fixations, could be an informative complement to behavioral responses on TOMM-C and that oculomotor measures are less vulnerable to coaching than behavioral responses.

## Materials and methods

### Subjects

**Figure 1:**
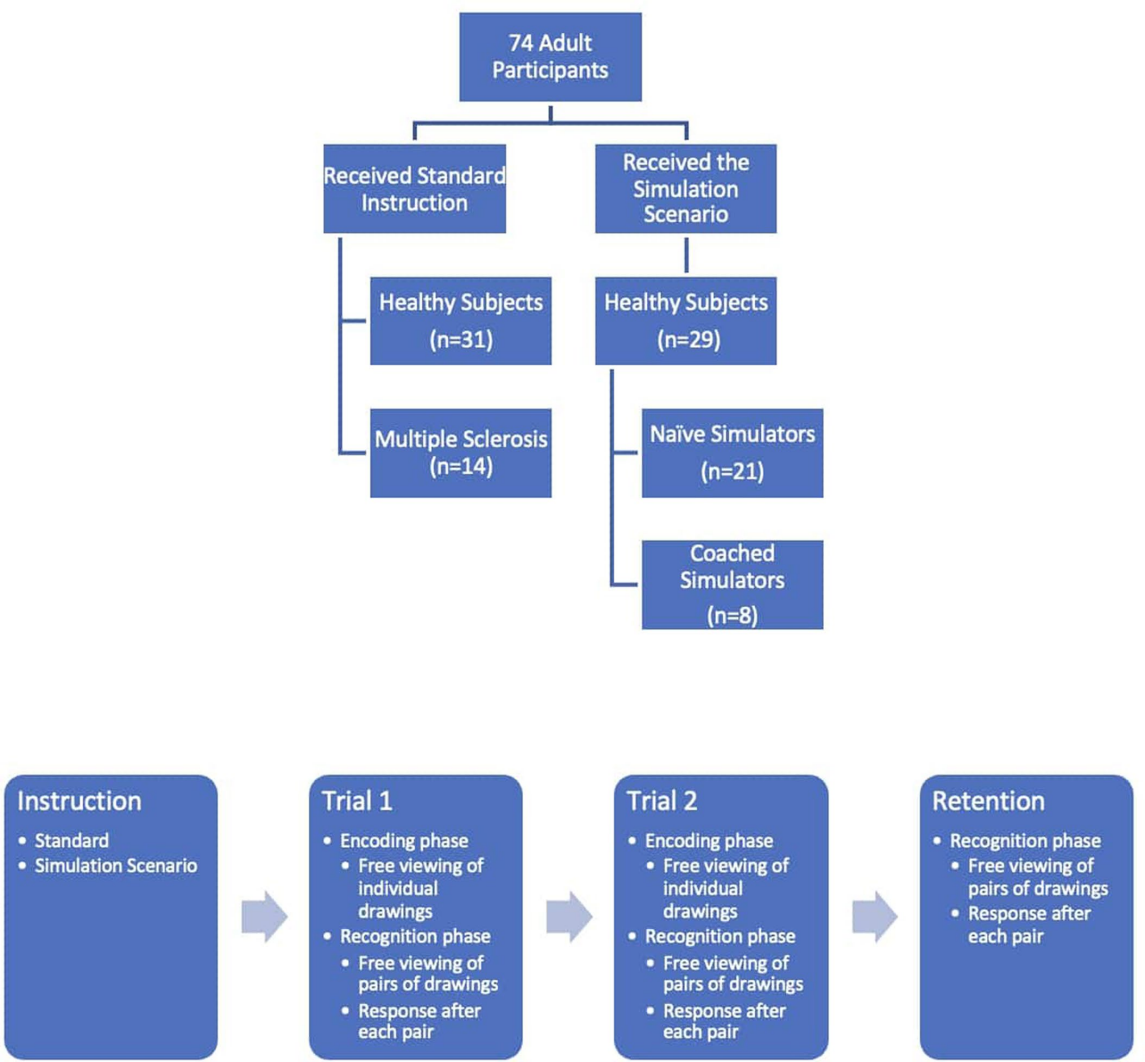
Flowchart of the study participants and of the TOMM experiment.

Sixty healthy subjects recruited in the community were asked to perform a computerized version of TOMM (TOMM-C). The participants were distributed into two groups: 31 healthy subjects received the standard instruction (SI group) and 29 were instructed to feign memory impairment as if they were in the initial stages of dementia to benefit from Social Security disability (21 were “Naïve Simulators” – NS group, and 8 were “Coached Simulators” – CS group). Fourteen patients with diagnosis of Multiple Sclerosis (Polman et al., 2011) and with cognitive complaints on the routine neurological consultation, but without history of optic neuritis, were recruited from the outpatient clinic (MS group). All participants provided their informed written consent in accordance with the Declaration of Helsinki and the Centro Hospitalar Universitário de Santo António’s Ethical Committee (reference number 026-DEFI/049-CES; Figure 1).

### Procedures

#### TOMM-C

**Figure 2:**
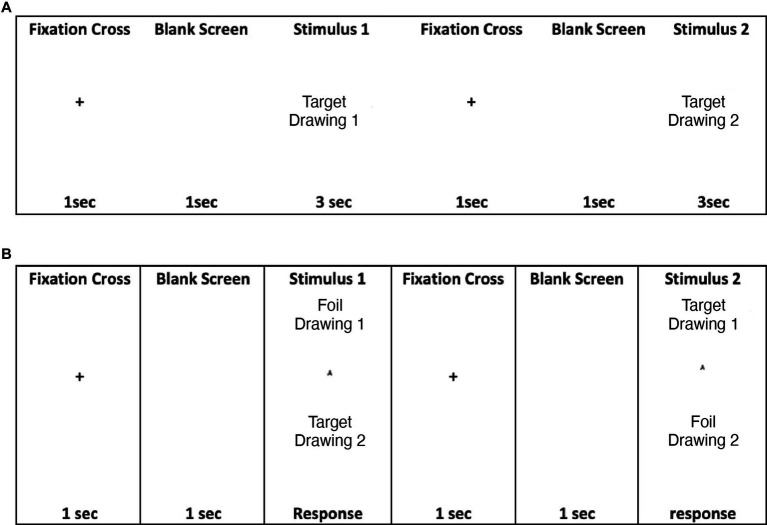
**(A)** TOMM encoding phase at Trials 1 and 2; **(B)** TOMM two choice recognition phase at Trial 1, Trial 2, and Retention.

TOMM-C is a computerized version of the standard TOMM (Tombaugh, 1996) adapted for eye-tracking recording. TOMM-C was presented in a Windows Based Software (Presentation® - Neurobehavioral Systems, Inc.). The stimuli were presented in a TFT Monitor 19″ with touch screen (KTMT-1921-USB/B, Keytec) with behavioral response recording. The iView X™ HiSpeed 1250 System (SensoriMotoric Instruments), with chin rest and forehead rest at 45 cm from the screen, eye-movements were recorded (i.e., monocular recording) during test performance. Similar to the standard version, TOMM-C is composed of two learning trials (Trial 1 and Trial 2) and a Retention trial. In both learning trials, there was an encoding phase and a recognition phase (Figure 2). During the encoding phase, participants were shown a series of simple line drawings (i.e., the same set of stimuli as the standard TOMM; Tombaugh, 1996) for 3 s each. Between items, a cross was displayed for 1 s on the screen followed by a blank screen for 1 s. The encoding phase was immediately followed by a two-choice recognition task. A Retention Trial, which was composed by just the two-choice recognition task, was administered following a 15-min delay (without further exposure of stimulus items). During the recognition phase of the three trials, after 3 s of free viewing of each pair of drawing, participants were cued by a buzz to respond with a touch on the screen (i.e., to identify the previously seen drawing). After the subject responded, a cross was displayed for 1 s on the screen followed by a blank screen for 1 s before the display of the next item. No feedback on the accuracy of response was provided. The pattern of eye-fixations was recorded during the free viewing of the test phase. The threshold to be considered a fixation was set at 100 ms (Manor and Gordon, 2003). Two areas of interest were identified: the “old” (i.e., drawing previously seen on the learning phase) and the “new” (i.e., foil drawing). Three behavioural measures were recorded: Number of Correct Responses, Total Response Time, and Median Response Time on Correct Responses. Three oculomotor measures were considered: % of Total Number of Fixation on “new” items, % of Total Fixation Time on “new” items, and % of Fixation Time on “new” items for correct responses.

Eye-fixation data on Trial 2 and Retention Trial from two NS participants were discarded due to recording problems that resulted in extensive missing data, however their behavioral responses on those trials were analyzed. One NS participant did not produce correct responses on Trial 2, therefore the Median Response Time on Correct Responses and the % of Fixation Time on “New” for Correct Responses could not be calculated.

#### Standard instruction and multiple sclerosis

SI and MS participants were asked to perform the TOMM-C to the best of their ability. The MS group were also asked to perform the Nine Hole Peg Test, the Symbol Digit Modalities Test – SDMT and the Auditory Verbal Learning Test - AVLT. The SDMT (Sousa et al., 2021) and AVLT (Cavaco et al., 2015) were adjusted to the demographic characteristics of the subjects according to the available norms.

#### Naïve and coached simulators

Both NS and CS participants were read the a scenario in which they were asked to imagine experiencing real memory difficulties and feeling no longer competent to carry out their work; and to request disability benefits they would need to go through a neuropsychological assessment and convince the examiner of their disability by highlighting their memory difficulties in a credible way. Following the literature (Frederick and Foster, 1991; Rüsseler et al., 2008; Jones, 2017), the CS participants additionally received a series of suggestions to produce the most severe memory problems *without* making it too obvious to the examiner.

### Statistical analyses

Descriptive statistics and nonparametric tests (Chi-square test or Fisher’s exact test and Mann–Whitney test) were used for characterization and comparison of the groups. Effect sizes were calculated and interpreted as follows: 0.2 (small), 0.5 (medium), and 0.8 (large). Receiver operating characteristic (ROC) curves were applied to differentiate SI and NS participants on each measure and trial. The area under the curve was calculated. By design, PVTs prioritize specificity over sensitivity and it is recommended that PVTs have at least 90% specificity when applied to individuals with evidence of significant cognitive dysfunction (Sherman et al., 2020; Sweet et al., 2021). Therefore, the specificity was set at ≥ 90%. The sensitivity, positive predictive value and negative predictive value were calculated. The cut-off scores were then used to identify frequency of abnormal score in MS and CS groups.

Multiple logistic regression analyses were used to explore the association between abnormal TOMM score performance and group, while taking into consideration demographic characteristics. TOMM score (recoded according to the cut-off) was the dependent variable, whereas group (i.e., SI vs. MS), sex, age, and years of education were the independent variables. No variable selection was applied and basic assumptions were verified. Simple logistic regression analyses were used to explore in MS group the association between performance on the SDMT and AVLT and some TOMM-C measures.

## Results

### Groups characteristics

As presented in Table 1, the SI group (*n* = 31) and the NS group (*n* = 21) had similar demographic characteristics, namely sex, age, and education. SI group was younger than the MS group (*n* = 14; *p* = 0.018) and had fewer years of education than the CS group (*n* = 8; *p* = 0.031). NS individuals were younger (*p* < 0.001) and had more years of education (*p* = 0.011) than MS patients; and were younger than CS (*p* = 0.029) participants. No group differences were recorded regarding sex.

**Table 1:** Demographic characterization and TOMM-C behavioral and eye-fixation scores.

			Standard instruction (SI) group (*n* = 31)	Naive simulators (NS) group (*n* = 21)	SI vs. NS		Multiple Sclerosis (MS) group (*n* = 14)	SI vs. MS		NS vs. MS		Coached simulators (CS) group (*n* = 8)	SI vs. CS		NS vs. CS	
					*p*	Effect size		*p*	Effect size	*p*	Effect size		*p*	Effect size	*p*	Effect size
Sex	Female		27 (87.1%)	19 (90.5%)	>0.999	0.05	11 (78.6%)	0.659	0.11	0.369	0.17	6 (75.0%)	0.583	0.14	0.300	0.20
Age	Years		31 (28, 46)	30 (28, 33)	0.182	0.19	45 (37, 52)	0.018	0.35	<0.001	0.67	35 (30, 45)	0.465	0.12	0.029	0.40
Education	Years		16 (15, 18)	17 (16, 18)	0.185	0.18	15 (12, 17)	0.185	0.20	0.011	0.43	18 (17, 21)	0.031	0.35	0.077	0.33
TOMM-C	Correct responses	Trial 1	49 (46, 50)	25 (18, 33)	<0.001	0.84	49 (47, 50)	0.940	0.01	<0.001	0.84	27 (20, 29)	<0.001	0.70	0.941	0.01
		Trial 2	50 (50, 50)	28 (22, 36)	<0.001	0.94	50 (50, 50)	0.502	0.10	<0.001	0.86	33 (30, 38)	<0.001	0.94	0.171	0.25
		Retention trial	50 (50, 50)	26 (17, 34)	<0.001	0.94	50 (50, 50)	0.559	0.09	<0.001	0.86	25 (17, 28)	<0.001	0.94	0.732	0.06
	Total response time	Trial 1	80.6 (69.7, 103.4)	94.3 (82.1, 134.5)	0.031	0.30	110.5 (86.8, 130.2)	0.005	0.42	0.711	0.06	102.5 (95.9, 119.1)	0.020	0.37	0.591	0.02
		Trial 2	66.5 (56.7, 83.6)	97.6 (64.7, 132.6)	0.009	0.36	94.3 (76.8, 112.9)	0.005	0.42	0.893	0.02	88.7 (75.9, 114.0)	0.018	0.38	0.770	0.05
		Retention trial	59.9 (46.5, 80.1)	88.6 (66.8, 105.6)	<0.001	0.48	88.4 (64.8, 101.8)	0.003	0.44	0.736	0.06	84.9 (67.4, 101.6)	0.044	0.32	0.845	0.04
	Median response time for correct responses	Trial 1	1.5 (1.2, 1.8)	1.8 (1.4, 2.4)	0.031	0.30	1.9 (1.4. 2.2)	0.012	0.38	0.920	0.02	1.9 (1.6, 2.3)	0.014	0.40	0.807	0.05
		Trial 2	1.3 (1.0, 1.6)	1.8 (1.2, 2.6)	0.007	0.37	1.7 (1.5, 1.9)	0.008	0.39	0.576	0.09	1.6 (1.5, 1.9)	0.031	0.34	0.684	0.08
		Retention trial	1.1 (0.9, 1.5)	1.6 (1.3, 2.0)	<0.001	0.48	1.7 (1.1, 1.9)	0.009	0.39	0.662	0.07	1.7 (1.2, 2.0)	0.037	0.33	0.884	0.03
	% of Total number of fixation on “New”	Trial 1	43.9 (39.1, 49.6)	50.7 (49.1, 53.2)	<0.001	0.66	49.2 (42.2, 52.4)	0.056	0.29	0.114	0.27	51.7 (50.0, 54.3)	<0.001	0.59	0.591	0.10
		Trial 2	47.0 (42.4, 52.9)	52.2 (50.6, 55.3)	0.016	0.34	49.1 (48.3, 54.0)	0.135	0.22	0.248	0.20	51.5 (43.3, 54.0)	0.554	0.09	0.387	0.16
		Retention trial	44.9 (41.1, 54.0)	53.8 (48.5, 59.8)	<0.001	0.51	54.1 (47.1, 59.1)	0.008	0.40	0.576	0.10	54.6 (51.3, 59.0)	0.004	0.46	0.799	0.15
	% of Total fixation time on “New”	Trial 1	43.1 (38.1, 47.4)	51.3 (49.6, 53.1)	<0.001	0.70	48.1 (41.5, 52.2)	0.047	0.30	0.055	0.33	51.1 (48.2, 52.6)	<0.001	0.53	0.696	0.07
		Trial 2	45.7 (39.4, 53.2)	53.6 (49.8, 55.4)	0.009	0.36	50.6 (47.3, 55.6)	0.148	0.22	0.401	0.14	51.0 (40.6, 55.9)	0.531	0.10	0.309	0.19
		Retention trial	44.1 (38.6. 52.7)	55.3 (48.0, 59.7)	<0.001	0.53	53.4 (46.5, 59.7)	0.007	0.42	0.552	0.10	54.8 (51.4. 61.1)	0.003	0.47	0.760	0.06
	% of Fixation time on “New” for correct responses	Trial 1	42.3 (38.0, 46.8)	50.2 (45.4, 53.2)	<0.001	0.46	48.1 (41.3, 51.8)	0.050	0.29	0.312	0.17	47.7 (40.1, 53.0)	0.082	0.28	0.464	0.14
		Trial 2	45.7 (39.4, 53.2)	51.3 (47.3, 57.4)	0.084	0.24	50.6. (47.3, 55.6)	0.148	0.22	0.942	0.01	47.2 (31.0, 55.2)	0.972	0.01	0.367	0.17
		Retention trial	44.1 (38.6, 52.7)	52.4 (46.5, 62.0)	0.007	0.38	53.4 (46.5, 60.0)	0.007	0.40	0.916	0.02	54.7 (47.9, 57.7)	0.047	0.32	0.879	0.03

Group comparisons. Data are presented as frequencies (%) and medians (25th, 75th percentiles). Chi-square test (or Fisher’s exact), Mann–Whitney test, and effect sizes were applied for group comparison. One Naïve Simulator did not produce correct responses on Trial 2. Missing data: eye-fixation data of Trial 2 and Retention Trial of two Naïve Simulator.

MS patients median *T*-score (25^th^, 75^th^ percentiles) on the SDMT was 44.1 (31.0, 50.5). The median adjusted score (25^th^, 75^th^ percentiles) of the AVLT-Delayed Recall was −1.0 (−1.7, 0.2). These adjusted scores correspond to the number of standard deviations below/above the mean of the participant’s normal peers with the same sex, age, and education. Three MS patients (21.4%) scored below the estimated 18^th^ percentile on AVLT-Delayed Recognition for the demographic characteristics of each individual.

### TOMM-C performance

As shown in Table 1, the NS group produced fewer correct responses (large effect sizes), had longer total response time and median response time on correct responses (small effect sizes), higher % of total number of fixations on “new” and % of total fixation time on “new” stimuli (medium effect sizes for Trials 1 and Retention, and small effects for Trial 2), and % of fixation time on “new” stimuli for correct responses (small effect sizes on all trials) in comparison to the SI group on all TOMM-C trials.

In comparison to the SI group, MS participants were slower to respond (i.e., total response time and median response time for correct responses) on all trials (*p* < 0.001), but had similar number of correct responses. The effect sizes for the response time measures were relatively small. On both Trial 1 (*p* < 0.06) and Retention Trial (*p* < 0.01), the % of total number of fixations, the % of total fixation time on “new,” and the % of fixation time on “new” for correct responses was higher for the MS group than the SI group, with a small effect size. The MS group only differed from the NS group on the number of correct responses (all *p* < 0.001 and with large effect sizes). Neither response time nor oculomotor measures differed between MS and NS participants.

NS and CS groups did not differ on any of TOMM-C behavioral and oculomotor measures. As shown in Table 1, the comparison between SI group and CS group revealed significant differences on all measures, but only partially (i.e., not on all trials). Regarding the number of correct responses, a medium effect size was observed for Trial 1 and large effect sizes were recorded for Trials 2 and Retention. For both Total Response Time and Median Response Time for Correct Responses, the effect sizes were relatively small. Medium effect sizes were observed for Trial 1 on the % of Total Number of Fixation on “New” and % of Total Fixation Time on “New.” Small effect sizes were also recorded on these measures at Retention Trial.

Table 2 shows the best cut-off scores to differentiate SI and NS participants, while setting the specificity at >90%. These cut-off scores were then used to identify the frequency of abnormal scores in the MS group and CS group.

**Table 2:** Diagnostic statistics of TOMM-C behavioral and eye-fixation measures, in the comparison between healthy with standard Instruction (SI) and healthy with naïve simulation instruction (NS) groups.

			Heathy with standard instruction vs. Healthy with naïve simulation instruction								Frequency of abnormal score	
			AUC	95% CI	*p*	Cut-off Point	Sensitivity	Specificity	Positive predictive value	Negative predictive value	Multiple sclerosis with standard instruction (MS) group (*n* = 14)	Healthy with coached simulation instruction (*n* = 8)
TOMM-C	Correct responses	Trial 1	0.993	0.979, 1.000	<0.001	45	93.5%	100%	91.3%	100%	0 (0%)	8 (100%)
		Trial 2	1.000	1.000, 1.000	<0.001	49	100%	100%	100%	100%	0 (0%)	8 (100%)
		Retention trial	1.000	1.000, 1.000	<0.001	49	100%	100%	100%	100%	0 (0%)	8 (100%)
	total response time	Trial 1	0.677	0.523, 0.832	0.031	122.5	33.3%	90.3%	70.0%	66.7%	5 (35.7%)	1 (12.5%)
		Trial 2	0.716	0.566, 0.865	0.009	97.0	52.4%	90.3%	78.6%	73.7%	7 (50.0%)	2 (25.0%)
		Retention trial	0.786	0.658, 0.915	0.001	100.6	33.3%	93.5%	77.8%	67.4%	4 (28.6%)	2 (25.0%)
	Median response time for correct responses	Trial 1	0.677	0.517, 0.838	0.031	2.1	28.6%	93.5%	75.0%	65.9%	5 (35.7%)	3 (37.5%)
		Trial 2	0.724	0.577, 0.872	0.007	1.8	50.0%	90.3%	76.9%	73.7%	5 (35.7%)	3 (37.5%)
		Retention trial	0.785	0.658, 0.912	0.001	2.0	23.8%	93.5%	71.4%	64.4%	2 (14.3%)	1 (12.5%)
	% of Total number of fixation on “New”	Trial 1	0.890	0.805, 0.975	<0.001	50.1	61.9%	96.8%	92.9%	78.9%	6 (42.9%)	6 (75.0%)
		Trial 2	0.739	0.602, 0.875	0.005	55.8	15.8%	90.3%	50.0%	63.6%	3 (21.4%)	0 (0%)
		Retention TRIAL	0.806	0.688, 0.925	<0.001	57.8	26.3%	90.3%	62.5%	66.7%	4 (28.6%)	2 (25.0%)
	% of Total fixation time on “New”	Trial 1	0.916	0.836, 0.995	<0.001	49.2	81.0%	93.5%	89.5%	87.9%	7 (50.0%)	6 (75.0%)
		Trial 2	0.756	0.623, 0.888	0.003	58.8	10.5%	90.3%	40.0%	62.2%	0 (0.0%)	1 (12.5%)
		Retention trial	0.818	0.704, 0.933	<0.001	57.8	35.0%	93.5%	77.8%	70.7%	4 (28.6%)	3 (37.5%)
	% of Fixation time on “New” for correct responses	Trial 1	0.774	0.636, 0.913	0.001	49.7	52.4%	93.5%	84.6%	74.4%	6 (42.9%)	3 (37.5%)
		Trial 2	0.683	0.534, 0.832	0.034	58.6	16.7%	90.3%	50.0%	65.1%	0 (0.0%)	1 (12.5%)
		Retention trial	0.730	0.580, 0.880	0.007	57.8	26.3%	93.5%	71.4%	67.4%	4 (28.6%)	2 (25.0%)

Frequency of abnormal score for the multiple sclerosis with standard instruction (MS) group and the healthy with coached simulation (CS) groups. AUC, the area under de curve. For all measures, except correct responses, scores > the cut-off point were considered abnormal. Missing data: Eye-fixation data on Trial 2 and Retention Trial from two Naïve Simulators were discarded due to recording problems that resulted in extensive missing data. One Naïve Simulator did not produce correct responses on Trial 2, therefore the Median Response Time on Correct Responses and the % of Fixation Time on “New” for Correct Responses could not be calculated.

Multiple logistic regression analyses revealed that, while taking into consideration demographic characteristics, MS patients had higher odds of abnormal score than SI participants on Total Response Time and Median Response Time for Correct Responses at Trial 1 (respectively adjusted odds = 6.441, *p* = 0.076 and adjusted odds = 25.027, *p* = 0.016) and Trial 2 (respectively adjusted odds = 11.001, *p* = 0.008 and adjusted odds = 4.476, *p* = 0.086). MS patients also had higher odds of abnormal score at Trial 1 on the following oculomotor measures: % of Total Number of Fixation on “New” (adjusted odds = 44.085, *p* = 0.005), % of Total Fixation Time on “New” (adjusted odds = 34.961, *p* = 0.003), and % of Fixation Time on “New” for Correct Responses (adjusted odds = 40.412, *p* = 0.007). No statistically significant difference (*p* > 0.05) on oculomotor measures was found between SI and MS participants on Trial 2 and Retention trial, when demographic characteristics were considered.

Simple logistic regressions were used to explore in MS patients the association between standard neuropsychological measures (i.e., SDMT and AVLT) and the following TOMM-C measures: Total Response Time, Median Response Time for Correct Responses, and % of Total Fixation Time on “New.” No significant association was found (*p* > 0.05).

## Discussion

Study results revealed that both behavioral responses (i.e., response accuracy and response time) and eye-fixation data can distinguish simulators from non-simulators in a computerized version of TOMM. Healthy simulators were asked to imagine experiencing “real” memory problems and needing to exaggerate their cognitive difficulties to obtain disability benefits.

Eye-fixation recordings of the SI group showed a familiarity preference (i.e., shorter fixation time on “new” stimuli), especially on Trial 1, whereas both simulator groups showed a novelty preference (i.e., longer fixations on “new” stimuli than on previously presented stimuli) on the three TOMM-C trials. The eye-fixation measure with the best diagnostic statistics in differentiating SI from NS participants was % of Total Fixation Time on “New” (sensitivity of 81.0% and specificity of 93.5%). These findings are consistent with a recent study (Tomer et al., 2020), which revealed that simulators spent more time gazing at foils than target stimuli in another PVT - the Word Memory Test. In non-clinical samples, a novelty preference appears to be a marker of non-credible performance on PVTs that require forced-choice recognition. It has also been suggested that visual disengagement (i.e., gaze aversion) may be used by simulators to attenuate visual input and thereby decrease the cognitive load that they may be experiencing while performing the test (Tomer et al., 2020). Though, gaze aversion could not be documented in the present study, because only two areas of interest - AOI (i.e., the screen was divided in two - “old”/ “new” drawings) were considered, fixations in non-relevant spaces within each AOI were considered on target, and fixations outside the two AOI were discarded. Future studies ought to explore in greater detail the viewing pattern during the performance of TOMM.

Forced-choice memory recognition PVTs (e.g., TOMM and Word Memory Test) share some resemblance with visual-paired comparison (VPC) tasks, which were designed to measure infant recognition memory. Both typically involve a familiarization phase followed by a test phase. During the familiarization phase, the individual is presented with a set of visual stimuli. During the test phase, the familiarization stimulus is paired with a novel stimulus. On VPC tasks, the spontaneous eye-movements are recorded and the amount of time spent looking at each stimulus during the test phase is usually the primary dependent variable. A decreased attention to familiar patterns relative to novel ones (i.e., spending more time looking at novel images) has been observed in VPC applied to preverbal human infants (Fantz, 1964), human adults (Manns et al., 2000), and primates (Pascalis and Bachevalier, 1999). VPC may also elicit a preference for familiarity depending on the length of the retention interval (Bahrick and Pickens, 1995; Richmond et al., 2007). Unlike standard VPC tasks, forced-choice memory recognition tests require an explicit recognition instruction and the visual behaviour of healthy adults during the test phase has been shown to favour familiar stimuli (Richmond et al., 2007; Brooks et al., 2023). Both the preference for novelty and the preference for familiarity are usually interpreted as evidence of recognition memory, whereas null preferences can be interpreted as evidence of forgetting (Richmond et al., 2007).

MS patients exhibited a less evident familiarity preference on the eye-fixation data than the SI participants on Trial 1. It’s unclear why half of the patients with MS showed a preference for the “New” stimuli, as measured by the % of Total Fixation Time on “New.” Both the preference for novelty and the preference for familiarity are usually interpreted as evidence of recognition memory, whereas null preferences can be interpreted as evidence of forgetting (Richmond et al., 2007). It is reasonable to speculate that MS patients were more alert to the possibility that novel stimuli might be relevant, because of their prior experience with neuropsychological assessments (for clinical purposes) that require recall and recognition of previously presented stimuli without prior warning. In the MS group, the % of Total Fixation Time on “New” was not related to measures of visual working memory/psychomotor speed (i.e., SDMT) and verbal memory (i.e., AVLT Delayed Recall and Delayed Recognition), even though patients’ performance on these standard neuropsychological measures was as expected mildly below the norm (Martins Da Silva et al., 2015). Future studies ought to explore the preference for familiarity / novelty in *bona fide* MS patients and in other clinical populations and to investigate their associations with standard measures of memory (both visual and verbal).

The number of Correct Responses produced the most robust diagnostic statistics and the identified cut-off scores are consistent with most studies in the literature that explored simulation in healthy individuals (for a review see: Martin et al., 2020). Healthy individuals feigning memory impairment significantly produced fewer correct responses on TOMM-C than the SI group. NS and CS performance on TOMM-C approached chance level, especially on Trial 1 and Retention Trial. A ceiling effect was observed in healthy individuals with credible performance (Rees et al., 1998). The number of Correct Responses was similar between MS patients and SI healthy individuals on all trials. Furthermore, only the number of Correct Responses clearly differentiated MS patients from NS participants. These results provide support to its use in clinical practice, namely in patients with MS, cognitive complaints, and mild memory difficulties.

Response time differentiated SI participants from both simulator groups, confirming previous reports (Bolan et al., 2002; Kanser et al., 2019) that healthy simulators are slower to respond on TOMM. However, MS patients were also slower to respond than healthy individuals under SI condition and had similar latency to the NS group. These results highlight the need for caution when applying response time as a performance validity measure in clinical populations, namely in MS which is known to produce processing speed deficits in most patients (Ruano et al., 2017). Nonetheless, in the present study no clear association was found between response time on TOMM-C and a standard measure of visual working memory and psychomotor speed (i.e., SDMT).

No effect of coaching how to avoid detection of invalid performance was observed on any of the behavioural and eye-fixation measures of TOMM-C. In other words, the performance of NS and CS participants did not differ, which may reflect lack of statistical power or resistance of the test to coaching (Jelicic et al., 2011). Larger samples are necessary to confirm these negative findings.

The simultaneous recording of both behavioral and eye-fixation measures in one of the most widely used PVTs, the exploration of different experimental conditions, and the inclusion of a clinical sample are strengths of the study. Unfortunately, the inclusion of participants was cut short due to equipment failure. As ensuing, the small size of the studied groups and the demographic differences of the groups (namely regarding age and education) limit the informative value of group comparisons and the generalizability of the research findings. Though, the literature has recorded minimal or no effects of age or education on TOMM performance (Rees et al., 1998; Rai and Erdodi, 2021; Tchienga and Golden, 2022). Furthermore, the characteristics of the clinical group were not ideal, because none of the MS participants with cognitive complaints had a diagnosis of dementia and not all had memory impairment. Future studies ought to study other clinical aetiologies and suspected clinical malingers.

Recent studies with pupillometry have reported that pupil dilation can detect feigned cognitive impairment on TOMM (Heaver and Hutton, 2011; Patrick et al., 2021a,b). However, the present study focused only on eye-fixations. Future studies should explore the possibility of combining pupil reactivity with eye-fixation pattern in the detection of deception. The standardization of the viewing period (3 s) prior to the behavioral response facilitated the comparison between participants, though it also limited the informative value of the response time.

In sum, healthy individuals feigning memory impairment showed a distinct behavioral (i.e., fewer correct responses and longer response times) and oculomotor (i.e., longer fixation time on “new” stimuli) response pattern on a computerized version of TOMM, which may reflect an increased effort to inhibit a natural response. Further investigation is necessary to understand the potential application of response time and eye-fixation measures in real-life clinical situations.

## Data availability statement

The raw data supporting the conclusions of this article will be made available by the authors, without undue reservation.

## Ethics statement

The studies involving humans were approved by Comissão de Ética do Centro Hospitalar Universitário de Santo António. The studies were conducted in accordance with the local legislation and institutional requirements. The participants provided their written informed consent to participate in this study.

## Author contributions

FG: Writing – original draft, Writing – review & editing, Investigation. IF: Investigation, Writing – review & editing, Writing – original draft. BR: Software, Writing – review & editing, Writing – original draft. AM: Methodology, Writing – review & editing, Writing – original draft. SC: Methodology, Funding acquisition, Supervision, Writing – original draft, Writing – review & editing.
