# Supplementary material for: Using behavior and eye-fixations to detect feigned memory impairment
Source: Front Psychol. 2024 Sep 20;15:1395434. doi: 10.3389/fpsyg.2024.1395434 (PMC11450296; doi:10.3389/fpsyg.2024.1395434)
Supplement: Supplementary file 1 [file Table_1.DOCX]

Both NS and CS participants were read the following scenario:

*“Imagine that lately you have been forgetting things a little more than usual and you are worried because you think it could be the beginning of a dementia process, for example Alzheimer’s disease. You no longer feel competent to carry out your work as you have done up until now. To request your Social Security disability, you need to be evaluated by a professional with a lot of experience in the area. In this way, you will undergo a neuropsychological assessment, in order to assess your real cognitive abilities, particularly memory. In order for your request to be accepted, you will try to highlight these difficulties throughout the assessment, in a credible way without the examiner being suspicious. Obvious exaggerations will be easy to detect and could penalize you in the final decision.”*

NS individuals then received the following instruction before performing the tests:

*“Please demonstrate to the technician examining you that you have serious difficulties in carrying out the requested task. Do it in a way that the technician considers credible and does not suspect your performance. Did you understand?”*

After the presentation of the scenario, CS participants received the following instruction:

*“Please demonstrate to the technician examining you that you have serious difficulties in carrying out the requested task, particularly in remembering what is asked of you. To this end, we suggest you use some of the following strategies: be slower in responding, respond correctly to the easiest items, make mistakes on the most difficult items, and answer at least half of the items correctly. It may help to know that many people with memory difficulties tend to experience the following symptoms: headaches, difficulties with concentration and understanding, tiredness and low tolerance to effort, and irritability, anxiety and/or depression. Do you understand what you have to do? Do you have any questions?”*
